# Factors Affecting Cellular Uptake of Anthocyanins: The Role of pH, Glucose and Anthocyanin Structure

**DOI:** 10.3390/nu14224807

**Published:** 2022-11-13

**Authors:** Yana Cahyana, Charlotte Elizabeth Mills, Syamsul Huda, Michael H. Gordon

**Affiliations:** 1 Food Technology Department, Faculty of Agricultural Industrial Technology, Universitas Padjadjaran, Jl. Raya Bandung Sumedang KM 21, Jatinangor 40600, West Java, Indonesia; 2 Hugh Sinclair Unit of Human Nutrition, Department of Food and Nutritional Sciences, School of Chemistry, University of Reading, Whiteknights, P.O. Box 226, Reading RG6 6AP, Berkshire, UK

**Keywords:** anthocyanins, structure, caco-2 cells, glucose, uptake, pH

## Abstract

Anthocyanins have poor bioavailability, but the factors affecting this remain unclear. Uptake into cells could impact the bioavailability; therefore, understanding factors affecting anthocyanin uptake is pivotal to improve their bioavailability and reveal the mechanism for their uptake. This study aimed to investigate the effect of anthocyanin structure, pH and glucose on the uptake of anthocyanins by Caco-2 cells. Anthocyanin extract from strawberry and red grape at 10 or 20 µM was added to Caco-2 cells. Anthocyanin toxicity to the cells was firstly examined to ensure the same cell viability. The uptake was carried out at pH 7 and 6.5 to evaluate the effect of pH. Glucose (1 mM) was used to investigate its effect. The results show that anthocyanins toxicity was dependent on the concentration and length of exposure. Anthocyanin uptake was concentration-dependent and affected by their structures, in which cyanidin-3-glucoside uptake was higher than pelargonidin-3-glucoside. No metabolites from Caco-2 cell activity were detected. An increased uptake with a decrease in pH was observed, which may be linked to the increase in anthocyanins stability and may indicate the role of proton co-transporter. This also suggests that the jejunum would be the favourable section of small intestine for anthocyanin uptake. Reduced anthocyanin uptake in the presence of glucose suggested that facilitative glucose transporter could be involved in the uptake of anthocyanins by Caco-2 cells.

## 1. Introduction

Anthocyanins and other flavonoids extracted from plum, peach, raspberry, and other sources such as vegetables, grains, tea have been identified to exhibit bioactive property [1,2,3,4,5]. This bioactive property has been demonstrated to provide beneficial effect on neuroprotection by reducing reactive oxygen species (ROS), increasing antioxidant enzyme activities [6] and cardio-health promotion [7]. However, literature reports that flavonoids, including anthocyanins, have poor bioavailability [8,9,10,11]. This low bioavailability leads to reduced health effect of anthocyanins when consumed in our daily life due to their low concentration in plasma or functional site in our body. Therefore, bioavailability is a key part for a bioactive compound to engender its beneficial effect.

Understanding factors that affect anthocyanins uptake into cells is an integral part of understanding their bioavailability. There is little research focusing on the uptake of anthocyanins into cells [11,12,13] and even less detailing the factors affecting their uptake.

An investigation into the effect of glucose on anthocyanin absorption using animals provides some evidence for the role of glucose transporters in anthocyanin absorption [14]. Reports on the involvement of glucose transporter pathway in anthocyanin absorption through intestinal cell have been documented elsewhere with conflicting conclusions [15,16].

Various anthocyanins with different structures are present in fruits and vegetables. Furthermore, anthocyanin stability is known to depend on pH [17]. Therefore, apart from glucose, other factors such as pH and anthocyanin structure are worth further investigation. The aim of this study was to examine factors likely to affect the uptake into cells (Caco-2 cells) including anthocyanin structure, presence of glucose, and pH. In the present study, anthocyanin extract from strawberry and red grape was used because these fruits contain different structure of anthocyanins such as cyanidine-3 glucoside, pelargonidin-3 glucoside and malvidin-3 glucoside [8] so the effect of anthocyanins structure can be revealed.

Caco-2 cells were chosen because they have been successfully used as an in vitro model to study the absorption or uptake of drugs and flavonoids [12,18,19]. In culture Caco-2 cells are subject to spontaneous enterocytic differentiation and polarization and can be used as a model system for the small intestinal epithelium [20,21].

## 2. Materials and Methods

### 2.1. Chemical and Reagents

Acetonitrile, methanol, formic acid, hydrochloric acid, water (HPLC grade), MK-571, Amberlite XAD7-HP, anthocyanin standards (pelargonidin-3 glucoside, pelargonidin-3-rhamnosylglucoside, cyanidin-3-glucoside, delphinidin-3- glucoside, malvidin-3-glucoside, petunidin-3-glucoside, peonidin-3-glucoside), 4’,6-diamidino-2-phenylindole (DAPI) were purchased from Sigma-Aldrich, Gillingham, UK. Caco-2 cells were purchased from European Collection of Authenticated Cell Cultures (ECACC), Salisbury, UK. Concentrate of Strawberry was purchased from Cobell, Exeter, UK and red grape concentrate from Homebrew shop, Aldershot, UK. The other reagents were from Lonza, Basle, Switzerland.

### 2.2. Extraction

Strawberry or red grape concentrate (40 g or 50 g, respectively) was mixed into 100 mL of 1% aqueous formic acid followed by removing the solid by vacuum filtration with Whatman no 4 filter paper. The filtrate was extracted with ethyl acetate then loaded onto an amberlite XAD7-HP column 60 cm long, 2 cm diameter which had been washed with water followed by methanol to remove monomers trapped on the resin. Acidified water was subsequently passed through the resin to remove glucose and other polar compounds until no glucose was detected in the washing water. Finally, the anthocyanins were eluted with acidified methanol, followed by removal of solvent with a rotary evaporator at about 40 °C. This solution of anthocyanin extract was then stored at −80 °C for later experiments of cell viability and uptake.

### 2.3. Cell Cultures

Caco-2 cells were seeded at a density of 5 × 10^5^ cells in a Petri dish of 10 cm diameter with a volume of 10 mL, containing DMEM supplemented with 10% FBS, 1% NEAA and 1% penicillin and streptomycin. The cells were grown in an incubator with 95% oxygen, 5% carbon dioxide, 95% humidity at 37 °C. Caco-2 cells were used between passages 65–76. The medium was changed every 2–3 days. On day 14, the cells were ready for the uptake experiment. Prior to the study, the medium were removed, and the cells were rinsed with PBS.

### 2.4. Cell Viability

Prior to the uptake experiment, cytotoxicity effect of anthocyanins onto Caco-2 cells at different concentrations, pH and length of exposure was examined. This is important to ensure that the uptake experiment is carried out at the similar level of cell viability. Therefore, any responses of the uptake experiment results from either difference in pH, glucose or anthocyanin structure. To verify the effect of anthocyanin cytotoxicity, first of all, the cell suspension was seeded onto the inner wells of 96 well plates at a density of 10^6^ cells/mL with the volume of 50 µL, while medium not containing cells was added to the outer wells. The cells were then incubated for 24 h in an incubator at 37 °C, under 5% CO_2_, 95% O_2_ and 95% humidity. Following the incubation, the medium was removed from all wells in the plate, and subsequently 50 µL of anthocyanin solutions (extracts from red grape and strawberry) ranging from 0 to 50 µM in DPBS was added into the wells, and then incubated for either 1 or 2 h. Each anthocyanin concentration was added into 4 wells per plate (3 with and 1 without cells). After the incubation, DPBS was removed, and chilled methanol was added into the wells. The methanol was then removed and 100 µL of DAPI solution was added followed by incubation for 30 min at 37 °C prior to measurement in a microplate reader at excitation and emission wavelengths of 340 and 465 nm, respectively. The experiment was repeated triplicate.

### 2.5. Uptake Experiment

The effect of anthocyanin structure was examined using anthocyanin extract from strawberry at 10 and 20 µM while the effect of glucose was performed at 20 µM red grape anthocyanin extract in the presence of 1 mM glucose. Additionally, 10 µM strawberry anthocyanins at pH 6.5 and 7 was used to examine the effect of pH on anthocyanin uptake.

Briefly anthocyanin stock solution was diluted to a final concentration of 10 or 20 µM in DPBS. The anthocyanins (10 mL) were immediately added to the cells, and subsequently incubated at 37 °C for 1h. Following the incubation, DPBS containing anthocyanins was removed and the cells were washed with PBS three times. The anthocyanins taken up by the cells were extracted with 6% formic acid and scraped off for collection. The extract along with the scraped cells were then sonicated, followed by centrifugation at 4 °C, 1700× *g* for 10 min. The supernatant was filtered and dried under nitrogen. The dry anthocyanins were redissolved in 200 µL of aqueous formic acid containing 10% acidified methanol, and then filtered into the insert prior to HPLC analysis.

### 2.6. HPLC Analysis

HPLC was used to identify and quantify anthocyanins present in juices and cellular extract following the uptake experiment. Anthocyanin standards and their standard curves were used to identify and quantify different anthocyanin structure present in juices and cellular extracts by determining their area under curve. The analysis was performed using an Agilent 1200 series HPLC with quaternary pump and diode array UV-visible detector. Sample (50 µL) was injected onto a reverse phase column of Nova-pak@C18 4 µm, with dimensions of 4.6 × 250 mm (Waters) equipped with a guard column. The mobile phases were 1% aqueous formic acid as solvent A and acetonitrile as solvent B. The flow rate was 1 mL/min with a column temperature of 35 °C. Compounds were eluted with a gradient of 100% solvent A at 0 min, changing to 81% A and 19% B at 19 min, 68% A and 32% B at 45 min, then 0% A and 100% B at 52 min. Solvent B was maintained at 100% for 5 min, and returned to 100% solvent A and 0% solvent B at 60 min. Wavelengths of 280 and 520 nm were chosen to detect the anthocyanins.

### 2.7. LC-MS Analysis

Several peaks detected in HPLC analysis either in juice or cellular extracts were not able to be identified due to the lack of anthocyanin standards; therefore, LC-MS was applied to identify the compounds. The identification of peaks using LC-MS particularly was conducted to main peaks present in juice (such as in red grape anthocyanins) or new peaks present in cellular extract which might be present as metabolites. Analysis by LC-MS was carried out with the same column and mobile phase as in the HPLC analysis. The LC-MS system consisted of an Agilent 1100 HPLC and a Bruker MicrotofQ II high resolution time of flight (TOF) mass spectrometer. The analysis was in the positive ion mode at 180 °C with electrospray ionisation. Nebulizer pressure at 1 bar N_2_, with flow rate of N_2_ gas 8 L/min were used. The collision voltage was 10 eV, collision RF 210 Vpp, capillary voltage −4000 V. Data were collected in high resolution mode.

### 2.8. Statistical Analysis

Data were analysed with the PASW statistic 17 package (SPSS Inc., Chicago, IL, USA). Analysis of variance, followed by LCD was used to examine the effect of anthocyanin concentration on cell viability and the effect of anthocyanin structure on uptake. Other data were analysed by Student’s t-test. Differences were considered as significant where *p* < 0.05.

## 3. Results

### 3.1. Anthocyanins Toxicity

It is necessary to carry out the uptake experiment in the similar cell viability. The difference in the cell viability may result from the cytotoxicity of anthocyanins added onto cells during the uptake experiment. Therefore, anthocyanins toxicity to Caco-2 cells is important to verify prior to the uptake experiment. Factors likely affecting the cytotoxicity includes the effect of length of exposure, anthocyanin concentration and pH of milieu. These factors were examined, and the results are presented below.

#### 3.1.1. Effect of Length of Exposure and Concentration

**Figure 1:**
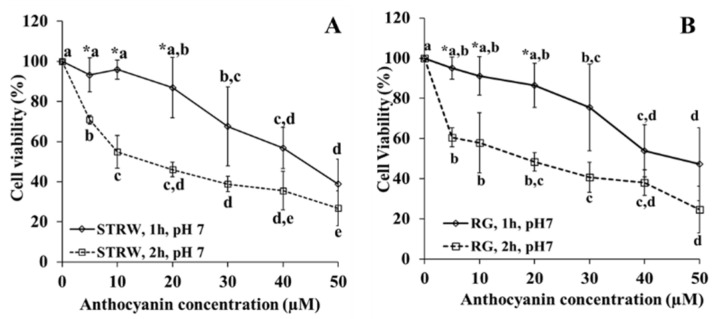
The effect of the concentration of strawberry (**A**) and red grape (**B**) anthocyanin on cytotoxicity at pH 7, incubation for 1 and 2 h at 37 °C. STRW = strawberry, RG = red grape. * Indicates significant difference (*p* < 0.05) for comparison at the same concentration but at different time of incubation. Lower case letters compare values for the same time of incubation at different concentration, with different letters in the compared values denoting significant difference at *p* < 0.05.

Cytotoxicity of anthocyanin extracts (Figure 1) is presented as a function of anthocyanin concentration and length of exposure (1 and 2 h of exposure) against cell viability (%). Both anthocyanins extracted from strawberry (A) and red grape (B) show cytotoxicity with the increase in anthocyanin concentration from 10 to 50 µM and of exposure length. A significant difference in cell viability was observed at 5 µM until 20 µM when comparing the effect of 1 h to 2 h exposure either in strawberry or red grape. Overall 1 h incubation results in higher cell viability than 2 h incubation. Therefore, it is important to choose the exposure for 1 h for the uptake experiment due to the higher cell viability.

At 1 h of exposure, no difference in toxicity was observed until 20 µM of anthocyanins either in strawberry or red grape. Consequently, to compare the extent of anthocyanin uptake from strawberry and red grape, the highest concentration at 20 µM was applied. For the effect of anthocyanin structure on the uptake, two concentrations at 10 µM and 20 µM of strawberry were employed during 1 h incubation.

#### 3.1.2. Effect of pH

**Figure 2:**
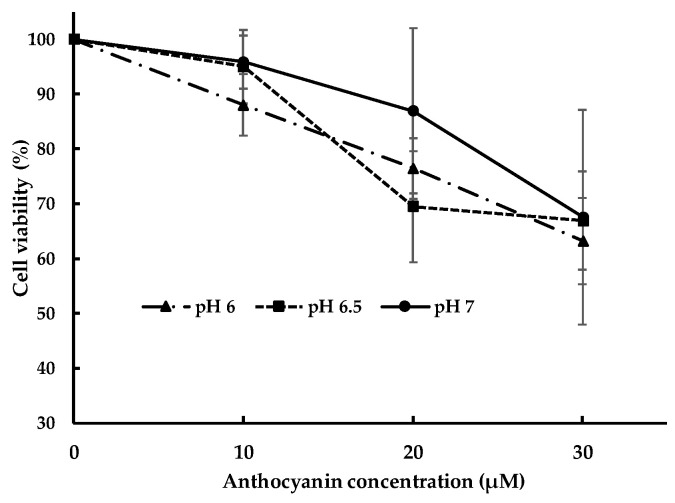
The effect of pH at different anthocyanin concentration on cytotoxicity.

The effect of pH of milieu on cell viability needs to be verified because it may affect anthocyanin toxicity to Caco-2 cells. The effect of pH on cell viability is presented in Figure 2. Cell viability of caco-2 cell at corresponding anthocyanin concentration was not significantly different at pH 7, pH 6.5 and pH 6. However, cell viability decreases with the increase in anthocyanin concentration at all pH. The value of cell viability at 10 µM at pH 7 and pH 6.5 were almost superimposed suggesting similar cell viability. For further examination of pH effect on anthocyanin uptake, treatment at pH 7 was compared to that at pH 6.5 using concentration at 10 µM; at this concentration the cell viability is the highest at approximately 95%.

### 3.2. Anthocyanin Taken Up by Cells

**Figure 3:**
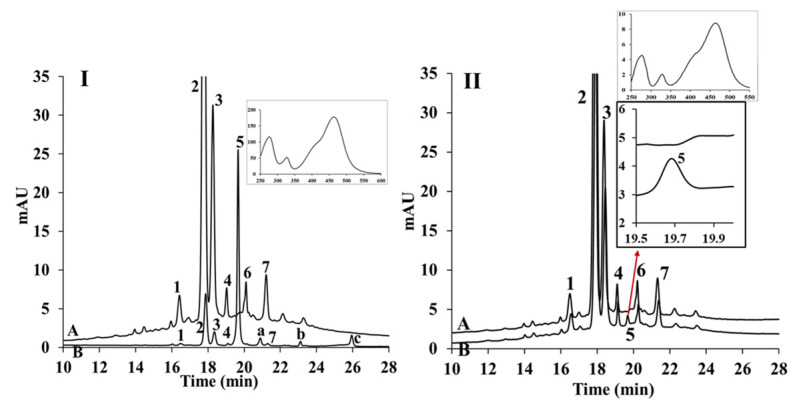
Chromatogram of strawberry juice extract; (**I**) describes uptake experiment (A) prior to cell uptake experiment, (B) extracted from cells following the uptake experiment. (**II**) describes the change of strawberry extract during 1 h incubation in the absence of the Caco-2 cells, (A) chromatogram of initial strawberry extract at t = 0 h; (B) chromatogram after 1 h incubation: 1 = cyanidin-3-glucoside; 2 = pelargonidin-3-glucoside; 3 = pelargonidin-3-rhamnosylglucoside; 4, 6, 7 = unknown compounds; Peaks 5, a, b, c =compounds appeared following uptake experiments. Inset is UV-visible spectrum of peak 5, λ_max_.= 275, 325 and 465 nm.

The strawberry juice used in this study contained cyanidin-3-glucoside, pelargonidin-3-glucoside and pelargonidin-3-rhamnosylglucoside with relative concentrations of 2.8%, 66.5% and 20.6%, respectively. Those anthocyanins were taken up by cells and detected by HPLC. Anthocyanins from strawberry at 20 µM was taken up by cells reaching 0.073% of initial concentration (Figure 3I). Several new peaks including those labelled as peaks 5, a, b, and c, corresponding to new compounds appeared in the cell extract. Peak 5 was the main compound present in the Caco-2 cell extract after incubation. Figure 3II presents the chromatogram of the anthocyanin extract before and after 1 h incubation in the absence of Caco-2 cells. Peak 5 appeared within 1 h incubation in the absence of cells, while peaks a, b, and c were not detected. After further investigation, peaks a, b, and c were identified using LC-MS.

According to HPLC analysis, red grape juice used in this study contained 5.7% delphinidin-3-glucoside, 10.3% cyanidin-3-glucoside, 9% petunidin-3-glucoside, 13.65% peonidin-3-glucoside, 39.76% malvidin-3-glucoside and two unknown compounds with quite big area under curve (peak 8 and 9) accounting for 2.5% and 9.1%, respectively. Therefore, the two compounds were identified using LC-MS. The compounds possessed m/z of 531/369 and 561/399.

**Figure 4:**
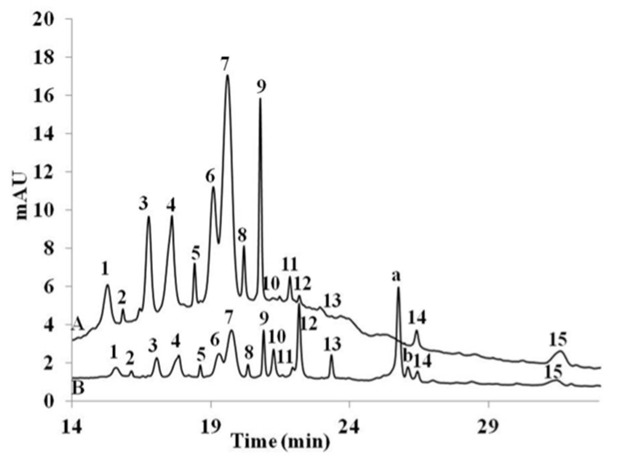
Red grape extract chromatogram (A) prior to cell uptake experiment, (B) extracted from cells following the uptake experiment: 1 = delphinidin-3-glucoside, 3 = cyanidin-3-glucoside, 4 = petunidin-3-glucoside, 6 = peonidin-3-glucoside, 7 = malvidin-3-glucoside, 8 = peonidin-3-glucoside pyruvic acid, 9 = malvidin-3-glucoside pyruvic acid; peaks 2, 5, 10, 11, 12, 13, 14 and 15 were unknown; peaks a, b appeared following uptake experiment.

Figure 4 shows the uptake of red grape anthocyanin by Caco2-cell monolayer reaching 0.051% of initial concentration. Ten percent of the compounds in red grape juice were unidentified. The 15 compounds initially present in the red grape juice were detected in the cell extract following the uptake study. Two new peaks a and b were present following 1h incubation.

### 3.3. Effect of Anthocyanin Structure on the Uptake

The effect of anthocyanin structure was examined using two concentrations of strawberry anthocyanins i.e 10 and 20 µM. Strawberry anthocyanins at 10 µM contain cyanidin-3-glucoside, pelargonidin-3-glucoside and pelargonidin-3-rhamnosylglucoside with the initial amount being 1.91, 45.69 and 14.50 µg/10mL, respectively. While the content in 20 µM was 3.89, 95.23 and 29.59 µg/10mL, respectively. Following the experiment of anthocyanin structure effect on the cellular uptake, cellular extracts were analysed using HPLC. Anthocyanins initially present in the strawberry juice were identified in the cellular extract and quantified. Cyanidin-3-glucoside, pelargonidin-3-glucoside and pelargonidin-3-rhamnosylglucoside initially present in 10 µM of strawberry juice was also present in cellular extract reaching 2.32 × 10^−2^, 2.88 × 10^−2^, and 2.66 × 10^−2^% of their initial concentrations, respectively. Meanwhile those in 20 µM of strawberry juice, the cellular extract contains 6.54 × 10^−^^2^, 6.95 × 10^−2^, and 5.93 × 10^−2^% of their initial concentrations, respectively. This result is tabulated in Table 1.

The effect of anthocyanins structure on the uptake was examined by extrapolating a given concentration (10 µg/10 mL) from two different experiments (10 and 20 µM) setting the intercept of the uptake in the absence of anthocyanin extract at zero. The extrapolated uptake at the same concentration is considered as normalised uptake. The result showed that anthocyanin uptake increases with the increase in initial anthocyanin concentration. For instance, at 1.91 µg/10 mL of initial concentration, the uptake cyanidin-3- glucoside was 2.32 × 10^−2^% of its initial concentration. The uptake increases to 6.54 × 10^−2^% of initial concentration when the initial concentration increases to 3.89 µg/10 mL. It suggests that the uptake of anthocyanins is concentration-dependent. Therefore, comparing the effect of structure on the uptake at the same initial concentraion is required to obtain proper result (normalised uptake). Result showed that normalised uptake of cyanidin-3-glucoside was the highest amongst the anthocyanins present in strawberry extract.

### 3.4. Effect of Glucose on Anthocyanin Uptake

**Figure 5:**
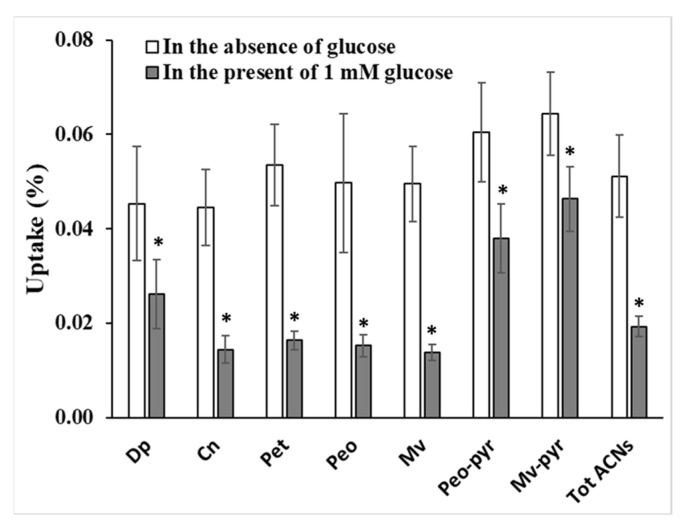
The influence of 1 mM glucose on the uptake of red grape anthocyanins by Caco-2 cells. Dp (delphinidin-3-glucoside), Cn (cyanidin-3-glucoside), Pet (petunidin-3-glucoside), Peo (peonidin-3-glucoside), Mv (malvidin-3-glucoside), Peo-pyr (peonidin-3-glucoside pyruvic acid), Mv-pyr (malvidin-3-glucoside pyruvic acid), Tot ACNs (total anthocyanins). * significant at *p* < 0.05.

The effect of glucose on the uptake of anthocyanins was carried out at 20 µM red grape in the presence of 1 mM glucose. Following the experiment, the cellular uptake was analysed and quantified using HPLC. Meanwhile LC-MS was used to identify 2 compounds present in red grape juice which are later known as peonidin-3-glucoside pyruvic acid and malvidin-3-glucoside pyruvic acid. The presence of glucose significantly decreased the uptake of all the anthocyanins in red grape juice compared to the control in the absence of glucose (Figure 5). Malvidin-3-glucoside followed with peonidin and petunidin-3-glucoside were the most sensitive to presence of glucose showing the uptake reduction by 69–72%. Uptake of cyanidin and delphinidin-3-glucoside decreased by 67 and 42%, respectively. The additional substitution of pyruvic acid into malvidin-3-glucoside significantly reduced the effect of glucose on anthocyanin uptake.

### 3.5. Effect of pH on Anthocyanin Uptake

**Figure 6:**
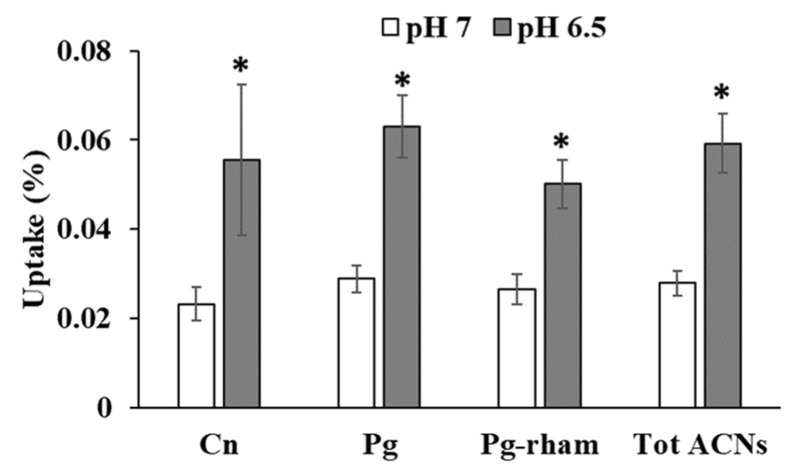
The increase in strawberry anthocyanin uptake with decreasing pH from pH 7 to 6.5 at a concentration of 10 µM anthocyanins. Cn (Cyanidin-3-glucoside), Pg (Pelargonidin-3-glucoside), Pg-rham (Pelargonidin-3-rhamnosylglucoside), Tot ACN (Total anthocyanins). * significant at *p* < 0.05.

The uptake of strawberry anthocyanins was significantly increased with decreasing the pH from 7 to 6.5 (Figure 6). Uptake of cyanidin-3-glucoside was the most sensitive to the pH change with an increase in uptake of 139%, followed by pelargonidin-3-glucoside with 118% increase in uptake. Pelargonidin-3-rhamnosylglucoside uptake was increased by 88%. The increase in the uptake of total strawberry anthocyanins was aproximately 112%.

## 4. Discussion

The anthocyanin addition onto Caco-2 cells may affect the cells viability due to the cytotoxicity effect. Therefore, it is pivotal to verify whether anthocyanins lead to a cytotoxicity effect. This information is also important to determine the anthocyanin concentration and the choice of pH for the anthocyanin uptake experiment. Ideally the chosen anthocyanin concentration is determined based on the highest anthocyanin concentration giving the highest cell viability.

The cytotoxicity experiment expressed in percentage of cell viability showed that anthocyanins at studied concentrations were toxic to Caco-2 cells. This finding was also demonstrated in another study on cytotoxicity of black carrot anthocyanin extract [22]. The toxicity was dependent on anthocyanin concentration and length of exposure. Comparing the effect of length exposure of 1 h to 2 h it is obvious that restriction of cell exposure to 1 h allowed concentrations up to 20 µM to result in similar cell viability for both the strawberry and red grape anthocyanins extracts. This concentration was the highest concentration with the highest and similar cell viability. Consequently, for the experiment of structure effect, concentrations of 10 and 20 µM were applied. Meanwhile for the experiment of glucose effect and the uptake of anthocyanins from strawberry and red grape, the highest concentration of 20 µM was used.

In terms of pH effect on cytotoxicity, the pH from 6–7 showed no different toxicity at corresponding anthocyanin concentration. To minimize the effects of cytotoxicity, the concentration of 10 µM at pH 7 and 6.5 was chosen for the experiment of the effect of pH on the anthocyanin uptake. At this concentration and pH, the cells had the highest and similar viability.

Analyzing anthocyanins from strawberry and red grape juice using HPLC it was found that the main anthocyanins present in strawberry were cyanidin-3-glucoside, pelargonidin-3-glucoside and pelargonidin-3-rhamnosylglucoside. These anthocyanins constitute approximately 90% of anthocyanins present in strawberry juice. Meanwhile red grape contains delphinidin-3-glucoside, cyanidin-3-glucoside, petunidin-3-glucoside, peonidin-3-glucoside, and malvidin-3-glucoside identified as the main anthocyanin (39.76%). Two main peaks in red grape juice was not identifiable by HPLC due to the lack of the anthocyanin standards. Therefore, LC-MS was applied. The two peaks exhibited m/z values of 531/369 and 561/399. These compounds were also glycosides, as indicated by the loss of 162 Da following fragmentation in the mass spectrum. These two compounds had the molecular formula C_25_H_23_O_13_ and C_26_H_25_O_14_, respectively suggesting that the compounds were peonidin-3-glucoside pyruvic acid and malvidin-3-glucoside pyruvic acid, respectively, also known as vitisin A compounds. Vitisin A along with vitisin B have been commonly found in red wine as a fermentation product. Although vitisin B has been previously found in grape juice [23]. In this study, we have also found vitisin A in red grape juice.

The uptake study using strawberry and red grape anthocyanin extracts demonstrated that anthocyanins are taken up by cells. Total uptake of red grape anthocyanins was smaller than that of strawberry (0.015% versus 0.073%) suggesting that uptake of anthocyanins in red grape are less favourable than those in strawberry. This finding may indicate the effect of anthocyanin structure on the extent of the uptake into cells. Several new peaks including those labelled as peaks 5, a, b, and c, corresponding to new compounds appeared in the cell extract. The literature includes conflicting reports about whether flavonoids, such as anthocyanins can be metabolised by Caco-2 cells, with metabolites found in one study [24] but not in a second study [25]. Anthocyanins are known to degrade rapidly at pH 7, so the possibility of the new peaks arising from chemical reaction during 1 h incubation was considered. In our case, peaks 5, a, b, and c were identified using LC-MS.

To assess degradation due to the pH, strawberry juice extract was incubated under the same conditions in the absence of the cells (Figure 3II). The appearance of peak 5 within 1 h incubation in the absence of cells suggested that the compound corresponding to peak 5 was formed by chemical reaction such as oxidation, cleavage and ring opening or reaction with other compounds during the incubation and was not a metabolite formed by the Caco-2 cells. Anthocyanins can form additional compounds important for copigmentation with phenolic acids [26] and can form pyranoanthocyanins [27].

The fact that peaks a, b, and c were not detected in the absence of the cells does not necessarily mean that these compounds are metabolites formed by the cells; it is possible that these compounds were present in small quantities in the cell extract that were not detected prior to incubation. In this experiment, the cell extract was concentrated to a great extent to boost the sensitivity of detection due to the low concentration of the compounds of interest, while in the absence of the cells, concentration of the sample was not carried out owing to the relatively high concentration of the compounds already present in the solution. Consequently, small concentrations of a component formed in the absence of the cells might not be detected.

Peaks a, b and c had molecular masses of 442, 486 and 316 Da with formulae C_19_H_38_O_11_, C_21_H_42_O_12_ and C_20_H_30_NO_2_, respectively. These masses did not correspond to expected metabolites of anthocyanins formed by glucuronidation, sulfoconjugation or methylation, leading to a conclusion that these peaks were not metabolites. The presence of a nitrogen atom in the molecular formula of peak c was an indication that this was not an anthocyanin derivative but probably an amino acid or inorganic nitrate already present in the juice.

The experiment testing different concentrations of strawberry anthocyanins showed that the percentage increase in uptake of anthocyanins into the cells increased of anthocyanin concentration (Table 1); demonstrating that the uptake was concentration-dependent and hence examining the effect of anthocyanin structure on the uptake should be estimated at the same concentration for each anthocyanin structure.

Amongst the anthocyanins present in the strawberry extract, cyanidin-3 glucoside was the most taken up by cells at the same initial amount of anthocyanin (10 µg/10 mL). The addition of rhamnosyl–glucoside to pelargonidin seems to favourably increase the uptake compared to the addition of glucoside. This finding demonstrates that anthocyanins structure affects the extent of anthocyanin uptake. Our finding is in line with that of another study highlighting the importance of anthocyanin structure in the uptake [11] which also found that cyanidin-3-glucoside was more taken up by cells than pelargonidin-3-glucoside. The fact that cyanidin-3-glucoside which is more polar than pelargonidin-3-glucoside was taken up more highly suggests that the uptake might not occur through the cell membrane by passive diffusion, as the cell membrane is hydrophobic in nature; the uptake might take place through facilitative transporter. The higher uptake of the more polar compound was also demonstrated in the higher uptake of total anthocyanins from strawberry than from red grape. The former predominantly contained pelargonidin-3 glucoside and malvidin-3 glucoside, respectively. These findings from this in vitro study is in agreement with our human intervention study where more anthocyanins excretion from strawberry was found in urine than from red grape [8].

The presence of 1mM glucose decreased the uptake of total red grape anthocyanins by 62%. The decrease in uptake in the presence of glucose indicates potential competition between glucose and anthocyanins for uptake into cells through the same transporter channels as previously described [28]. Our data indicate the possibility that the uptake of anthocyanins occur through apical membrane cells involving the glucose transporter pathway. This finding is in line with previous work demonstrating that absorption of anthocyanins through Caco-2 cells involves facilitative glucose transporters2 (GLUT2) [15] and also aligns with a human intervention study [29]. This concept is akin to the suggested uptake mechanism for flavonol glycosides [30,31]. This suggests the possibility of competition with other flavonoids in the uptake of anthocyanins when other flavonoids are present, particularly when the glucose transporter is saturated with these compounds.

The uptake competition amongst anthocyanins may be indicated from the different level of anthocyanin sensitivity to the glucose effect. In the absence of glucose, the more polar anthocyanins were more favourably taken up by cells. In other words, the high sensitivity of malvidin-3 glucoside to glucose compared to cyanidin and delphinidin-3 glucoside may relate to its lower uptake due to the less polar nature of malvidin.

The study investigating the impact of pH shows that pH of milieu affects the extent of anthocyanin uptake. The increased uptake with decreasing pH may be linked to the anthocyanins stability and indicate the involvement of pH dependent transport mechanisms involving a proton co-transporter. Observation of anthocyanins during a 1 h incubation indicated that stability of cyanidin-3 glucoside, pelargonidin-3 glucoside and pelargonidin-3 rhamnocylglucoside at pH 7 was 63.1, 67.7 and 72.3%, respectively and it increased to 88.7, 93.7 and 91.8% at lower pH of 6.5.

The small intestine consists of 3 sections namely the duodenum, jejunum and ileum in which the pH has been shown to progressively increase from about 5.5–6 in the duodenum to about 7–7.5 in the terminal ileum [32,33]. Therefore, anthocyanin absorption through the apical membrane could be much better in the upper part of the intestinal tract including the duodenum and jejunum than in the ileum.

The acidic pH studied in this experiment was closer to the environment of the jejunum. Considering the length of the duodenum which is only 5% of the length of the small intestine, it is reasonable to speculate that the jejunum, which is much longer than the duodenum (almost 50% of small intestinal length), is a more favourable site for anthocyanin uptake. Using our data for total anthocyanin at pH 6.5, uptake reached 479 pg/cm^2^ using 10 µM concentration of anthocyanins; the uptake of anthocyanins through the jejunum with surface area of 184 m^2^ with length of 280 cm [32] might reach as high as 0.88 mg in 1 h. In vivo trials have shown that the maximum concentration of anthocyanins in plasma is reached after about 1 h post ingestion, so uptake in 1 h is relevant. Absorption of 0.88 mg pelargonidin glucoside would lead to a blood concentration of 400 nM, or 727 nM in plasma, assuming a volume of 5 litres of blood. In an intervention study, the main metabolite, pelargonidin-O-glucuronide, reached a peak plasma concentration of 274 +/−24 nM after 1.1 +/−0.4 h when strawberries containing 222 µmol of pelargonidin-3-O-glucoside were ingested [34], so the calculation leads to concentrations of the right order of magnitude.

## 5. Conclusions

Anthocyanin cytotoxicity to the cells is dependent on anthocyanin concentration and length of exposure. Anthocyanins are taken up by Caco-2 cells with no detected metabolites being present. The anthocyanin uptake is concentration-dependent and affected by anthocyanin structure, pH of milieu and the presence of glucose. Cyanidin-3 glucoside which is more polar than pelargonidin-3-glucoside is more favourably taken up by the cells. The higher uptake of strawberry anthocyanins compared to red grape anthocyanins may be due to the more polar anthocyanins being present in strawberry (predominantly cyanidin-3-glucoside) compared to red grape which is mainly malvidin-3-glucoside. This structure effect may indicate that anthocyanin uptake may occur through facilitative transporter.

The decreased uptake of anthocyanins in the presence of glucose confirmed the role of facilitative transporter by glucose transporter. The sensitivity of anthocyanin uptake to the presence of glucose is structure-dependent with the less polar anthocyanins being the more sensitive. Therefore, in this context, glucose addition into anthocyanin juice might be not beneficial due to the reduced uptake of anthocyanins. The role of pH in anthocyanin uptake might be linked to the increase in anthocyanin stability and indicate the involvement of proton dependent co-transporters. It also suggests that anthocyanins could be better absorbed in the upper part of the small intestine (the jejunum) compared to the other parts of the small intestine.

## Figures and Tables

**Table 1:** The effect of anthocyanin structure on the uptake by Caco-2 cells.

Anthocyanins	Initial Amount (µg/10 mL)	Uptake (%)	Normalised Uptake (%)
Cn	0	0	
	1.91	2.32 × 10^−2^	
	3.89	6.54 × 10^−2^	
	10		0.159
Pg	0	0	
	45.69	2.88 × 10^−2^	
	95.23	6.95 × 10^−2^	
	10		0.007
Pg-rham	0	0	
	14.50	2.66 × 10^−2^	
	29.59	5.93 × 10^−2^	
	10		0.02

Pg (pelargonidin-3 glucoside), Cn (cyanidin-3-glucoside), Pg-rham(pelargonidin rhamnocylglucoside). Normalised uptake was calculated at 10 µg/10 mL derived from extrapolation of 3 different concentrations for a given anthocyanin

## Data Availability

Not applicable.

## References

[1] Chen X., Yang Y., Yang X., Zhu G., Lu X., Jia F., Diao B., Yu S., Ali A., Zhang H. (2022). Investigation of flavonoid components and their associated antioxidant capacity in different pigmented rice varieties. Food Res. Int..

[2] Perez M.B., Da Peña Hamparsomian M.J., Gonzalez R.E., Denoya G.I., Dominguez D.L.E., Barboza K., Iorizzo M., Simon P.W., Vaudagna S.R., Cavagnaro P.F. (2022). Physicochemical properties, degradation kinetics, and antioxidant capacity of aqueous anthocyanin-based extracts from purple carrots compared to synthetic and natural food colorants. Food Chem..

[3] Ali A., Cottrell J.J., Dunshea F.R. (2022). Identification and characterization of anthocyanins and non-anthocyanin phenolics from Australian native fruits and their antioxidant, antidiabetic, and anti-Alzheimer potential. Food Res. Int..

[4] Toshima S., Hirano T., Kunitake H. (2021). Comparison of anthocyanins, polyphenols, and antioxidant capacities among raspberry, blackberry, and Japanese wild Rubus species. Sci. Hortic..

[5] Shen N., Wang T., Gan Q., Liu S., Wang L., Jin B. (2022). Plant flavonoids: Classification, distribution, biosynthesis, and antioxidant activity. Food Chem..

[6] Zhang Y., Yin L., Huang L., Tekliye M., Xia X., Li J., Dong M. (2021). Composition, antioxidant activity, and neuroprotective effects of anthocyanin-rich extract from purple highland barley bran and its promotion on autophagy. Food Chem..

[7] Wang S., Melnyk J.P., Tsao R., Marcone M.F. (2011). How natural dietary antioxidants in fruits, vegetables and legumes promote vascular health. Food Res. Int..

[8] Cahyana Y., Gordon M.H., Gibson T.M. (2019). Urinary Excretion of Anthocyanins Following Consumption of Strawberry and Red Grape Juice. Int. J. Vitam. Nutr. Res..

[9] Gordon M.H. (2012). Significance of dietary antioxidants for health. Int. J. Mol. Sci..

[10] Alvarez-Suarez J.M., Cuadrado C., Redondo I.B., Giampieri F., González-Paramás A.M., Santos-Buelga C. (2021). Novel approaches in anthocyanin research—Plant fortification and bioavailability issues. Trends Food Sci. Technol..

[11] Sigurdson G.T., Atnip A., Bomser J., Giusti M.M. (2018). Aglycone structures and glycosylations affect anthocyanin transport and uptake in human gastric epithelial (NCI-N87) cells. J. Food Compos. Anal..

[12] Yi W., Akoh C.C., Fischer J., Krewer G. (2006). Absorption of Anthocyanins from blueberry extracts by Caco-2 human intestinal cell monolayers. J. Agric. Food Chem..

[13] Steinert R.E., Ditscheid B., Netzel M., Jahreis G. (2008). Absorption of black currant anthocyanins by monolayers of human intestinal epithelial Caco-2 cells mounted in ussing type chambers. J. Agric. Food Chem..

[14] Felgines C., Texier O., Besson C., Vitaglione P., Lamaison J.-L., Fogliano V., Scalbert A., Vanella L., Galvano F. (2008). Influence of glucose on cyanidin 3-glucoside absorption in rats. Mol. Nutr. Food Res..

[15] Faria A., Pestana D., Azevedo J., Martel F., de Freitas V., Azevedo I., Mateus N., Calhau C. (2009). Absorption of anthocyanins through intestinal epithelial cells—Putative involvement of GLUT2. Mol. Nutr. Food Res..

[16] Walton M.C., McGhie T.K., Reynolds G.W., Hendriks W.H. (2006). The flavonol quercetin-3-glucoside inhibits cyanidin-3-glucoside absorption in vitro. J. Agric. Food Chem..

[17] Castañeda-Ovando A., Pacheco-Hernández M.d.L., Páez-Hernández M.E., Rodríguez J.A., Galán-Vidal C.A. (2009). Chemical studies of anthocyanins: A review. Food Chem..

[18] Lennernäs H., Palm K., Fagerholm U., Artursson P. (1996). Comparison between active and passive drug transport in human intestinal epithelial (caco-2) cells in vitro and human jejunum in vivo. Int. J. Pharm..

[19] Liu Y., Hu M. (2002). Absorption and metabolism of flavonoids in the caco-2 cell culture model and a perused rat intestinal model. Drug Metab. Dispos..

[20] Hidalgo I.J., Raub T.J., Borchardt R.T. (1989). Characterization of the human colon carcinoma cell line (Caco-2) as a model system for intestinal epithelial permeability. Gastroenterology.

[21] Artursson P., Palm K., Luthman K. (2001). Caco-2 monolayers in experimental and theoretical predictions of drug transport. Adv. Drug Deliv. Rev..

[22] Aslan Türker D., Doğan M. (2022). Ultrasound-assisted natural deep eutectic solvent extraction of anthocyanin from black carrots: Optimization, cytotoxicity, in-vitro bioavailability and stability. Food Bioprod. Process..

[23] Wang H.B., Race E.J., Shrikhande A.J. (2003). Characterization of anthocyanins in grape juices by ion trap liquid chromatography-mass spectrometry. J. Agric. Food Chem..

[24] Murota K., Shimizu S., Chujo H., Moon J.H., Terao J. (2000). Efficiency of absorption and metabolic conversion of quercetin and its glucosides in human intestinal cell line Caco-2. Arch. Biochem. Biophys..

[25] Boyer J., Brown D., Liu R.H. (2004). Uptake of quercetin and quercetin 3-glucoside from whole onion and apple peel extracts by Caco-2 cell monolayers. J. Agric. Food Chem..

[26] Eiro M.J., Heinonen M. (2002). Anthocyanin color behavior and stability during storage: Effect of intermolecular copigmentation. J. Agric. Food Chem..

[27] Rentzsch M., Schwarz M., Winterhalter P. (2007). Pyranoanthocyanins—An overview on structures, occurrence, and pathways of formation. Trends Food Sci. Technol..

[28] Cahyana Y., Adiyanti T. (2021). Flavonoids as Antidiabetic Agents. Indones. J. Chem..

[29] Mulleder U., Murkovic M., Pfannhauser W. (2002). Urinary excretion of cyanidin glycosides. J. Biochem. Biophys. Methods.

[30] Wolffram S., Blöck M., Ader P. (2002). Quercetin-3-glucoside is transported by the glucose carrier SGLT1 across the brush border membrane of rat small intestine. J. Nutr..

[31] Walgren R.A., Lin J.T., Kinne R.K., Walle T. (2000). Cellular uptake of dietary flavonoid quercetin 4’-beta-glucoside by sodium-dependent glucose transporter SGLT1. J. Pharmacol. Exp. Ther..

[32] Balimane P.V., Chong S. (2005). Cell culture-based models for intestinal permeability: A critique. Drug Discov. Today.

[33] Evans D.F., Pye G., Bramley R., Clark A.G., Dyson T.J., Hardcastle J.D. (1988). Measurement of gastrointestinal ph profiles in normal ambulant human-subjects. Gut.

[34] Mullen W., Edwards C.A., Serafini M., Crozier A. (2008). Bioavailability of pelargonidin-3-O-glucoside and its metabolites in humans following the ingestion of strawberries with and without cream. J. Agric. Food Chem..

